# Cardiac imaging with photon counting CT

**DOI:** 10.1259/bjr.20230407

**Published:** 2023-10-24

**Authors:** Thomas Flohr, Bernhard Schmidt, Stefan Ulzheimer, Hatem Alkadhi

**Affiliations:** 1 Siemens Healthcare GmbH, Computed Tomography, Forchheim, Germany; 2 Diagnostic and Interventional Radiology, University Hospital Zurich, University of Zurich, Zurich, Switzerland

## Abstract

CT of the heart, in particular ECG-controlled coronary CT angiography (cCTA), has become clinical routine due to rapid technical progress with ever new generations of CT equipment. Recently, CT scanners with photon-counting detectors (PCD) have been introduced which have the potential to address some of the remaining challenges for cardiac CT, such as limited spatial resolution and lack of high-quality spectral data. In this review article, we briefly discuss the technical principles of photon-counting detector CT, and we give an overview on how the improved spatial resolution of photon-counting detector CT and the routine availability of spectral data can benefit cardiac applications. We focus on coronary artery calcium scoring, cCTA, and on the evaluation of the myocardium.

## Introduction

CT of the heart, in particular ECG-controlled coronary CT angiography (cCTA), has become clinical routine due to rapid technical progress with ever new generations of CT equipment.^1^ Comprehensive reports on the possibilities and clinical benefits of cardiac CT are available in review articles.^2–5^

Recently, CT scanners with photon-counting detectors (PCD) have been introduced which have the potential to address some of the remaining challenges for cardiac CT,^6^ such as limited spatial resolution and lack of high-quality spectral data. In this review article, we briefly discuss the technical principles of photon-counting detector CT (PCD-CT), and we give an overview of cardiac applications. Other reviews on PCD-CT can be found in McCollough et al, Sartoretti et al, Flohr et al, Greffier et al, Leng et al,^7–12^ and those with a special focus on cardiovascular imaging in Meloni et al, Sandfort et al and Zsarnóczay et al.^13–15^

## Basic principles of photon-counting detector CT

The technical characteristics of a PCD will not be described in detail here—interested readers are referred to Flohr et al, Greffier et al, Willemink et al and Taguchi.^10,11,16,17^

**Figure 1.**
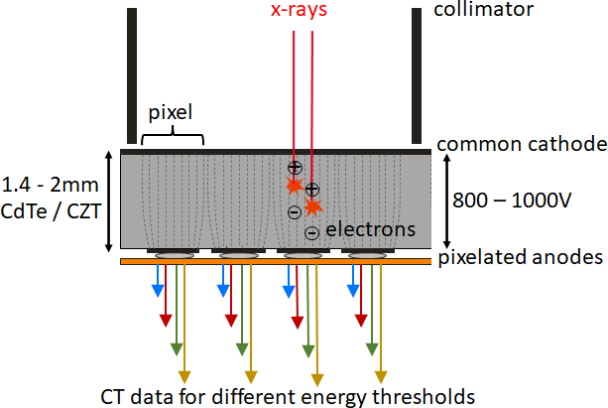
Schematic representation of a CdTe/CZT photon-counting detector. The detector pixels are formed by a strong electric field between the common cathode and the pixelated anodes (indicated by dashed lines) without demarcation by separating layers. In the detector structure shown here, there are four pixels between two collimator blades which serve to suppress scattered radiation. Four data streams per pixel (indicated by colored arrows) correspond to four different threshold energies for counting (and thus four different energy ranges of the registered X-rays). CdTe, cadmium telluride; CZT, cadmium zinc telluride.

A PCD consists of a semi-conductor such as cadmium telluride, cadmium zinc telluride, or silicon. The incident X-ray quanta are absorbed in this semi-conductor and converted directly into electrical signals (Figure 1). This is the essential difference to scintillation detectors previously used in medical CT, in which the X-ray quanta first generate visible light which is then converted into electrical signals.

The magnitude of the signal pulses in the semi-conductor is proportional to the absorbed energy of the X-ray quanta. The pulses are counted by electronics as soon as they exceed a threshold energy—hence the name “photon-counting detector”. By simultaneously applying several different thresholds, the registered X-ray quanta are divided into different energy ranges (Figure 1). PCDs can therefore routinely provide spectral CT data. Unlike scintillation detectors, PCDs are insensitive to electronic background noise, because the counting thresholds are far above the background noise level. Therefore, at low X-ray flux, CT images show better image quality with less image noise than those of scintillation detectors. Counting of all X-ray quanta, even those at lower energy, with the same weight leads to higher tissue contrasts in scans with iodinated contrast agent. Spatial resolution is substantially higher than with previous medical CT, because PCDs can be structured more finely than scintillation detectors—they do not require optical crosstalk to be prevented by separating layers between the individual detector elements. Conventional CT scanners provide isotropic spatial resolution of approximately 0.4–0.5 mm for cCTA, with the one exception of a device with smaller detector elements (Aquilion Precision, Canon Medical Systems, Japan).^18^ For a clinical dual source PCD-CT (NAEOTOM Alpha, Siemens Healthcare, Germany) a maximum spatial resolution in the scan plane of 0.208 mm for standard scans (144 × 0.4 mm collimation) and 0.125 mm for UHR scans (120 × 0.2 mm collimation) was demonstrated.^19^ The minimum slice width was 0.64 mm for a nominal 0.4 mm slice and 0.34 mm for a nominal 0.2 mm slice. Through-plane resolution corresponds to about half the slice width.^20^

## Coronary artery calcium scoring

The quantification of coronary artery calcium (CAC) to predict the likelihood of cardiovascular events was established in the early 1990s for electron beam CT (EBCT).^21^ A consensus standard for CAC scoring with multislice CT was developed in 2007.^22^ The scan and reconstruction parameters suggested therein (120 kVp X-ray tube voltage, 2.5–3 mm axial slice thickness) are now widely established. Nevertheless, there is still substantial variability in the Agatston scores measured with CT equipment from different vendors.^23^

Studies with PCD-CT prototypes on phantoms^24^ and cadaveric specimen^25^ have demonstrated that the established scan and reconstruction parameters can also be applied to PCD-CT and result in CAC scores comparable to conventional CT. PCD-CT was able to quantify CAC more accurately than conventional CT, in particular with reduced slice thickness^24^ or sharper kernels.^25^

Significant radiation dose reduction for CAC scoring compared to conventional CT was first demonstrated on volunteers with a PCD-CT prototype.^26^ In a study of 10 patients who underwent CAC scoring on both third-generation dual source CT and clinical PCD-CT with adjusted protocol parameters, comparable scoring results were obtained at half the radiation dose for PCD-CT (dose–length product (DLP) 38.7 mGy cm for PCD-CT *vs* 74.8 mGy cm for conventional CT).^27^

PCD-CT routinely provides virtual monoenergetic images (VMI) based on spectral material decomposition into iodine and soft tissue. On VMIs, the iodine attenuation (in Hounsfield units, HUs) is the same as if the image had been acquired with a monoenergetic X-ray beam of the desired energy (in keV), regardless of the actual X-ray tube voltage (in kVp) at data acquisition. This approach can lead to better standardization of image results.^28^ However, for CAC scoring, the appropriate keV must be chosen to reproduce the scoring results of conventional CT as closely as possible. In a phantom study of cadaveric hearts on a prototype PCD-CT, the best agreement was obtained at a VMI energy level of 72 keV for 120 kVp scans.^29^ Other authors found a high correlation between scores of a commercially available CAC scoring phantom obtained with clinical PCD-CT in VMIs at 70 keV without iterative reconstruction and those of conventional CT (both at 120 kVp), but PCD-CT yielded systematically slightly lower scores. These results were confirmed in 23 patients who had lower median Agatston scores on PCD-CT, but these were not statistically significant.^30^ Based on current knowledge, VMIs at 70 keV are generally recommended for CAC scoring with PCD-CT.

The choice of the optimal keV level for CAC scoring also depends on the use of iterative reconstruction.^31^ The results of a phantom study on a clinical PCD-CT^31^ suggest the possibility of compensating for decreasing CAC scores with increasing strength of iterative reconstruction compared to filtered backprojection by reconstructing VMIs at lower keV – then, iterative reconstruction which is not currently recommended for CAC scoring might be used to reduce radiation dose. The approach of approximating CAC scores of conventional CT by fine-tuning the keV-level of VMIs and the strength of iterative reconstruction was extended to PCD-CT with X-ray tube voltages other than 120 kVp for further radiation dose reduction. Use of 90 kVp and Sn100 kVp (this is 100 kVp with spectral shaping by a tin filter) with appropriately adjusted keV-level and iterative reconstruction resulted in CAC scores comparable to conventional CT,^32–34^ with radiation dose reduction between 19 and 67% depending on the study design and the reference dose.

**Figure 2.**
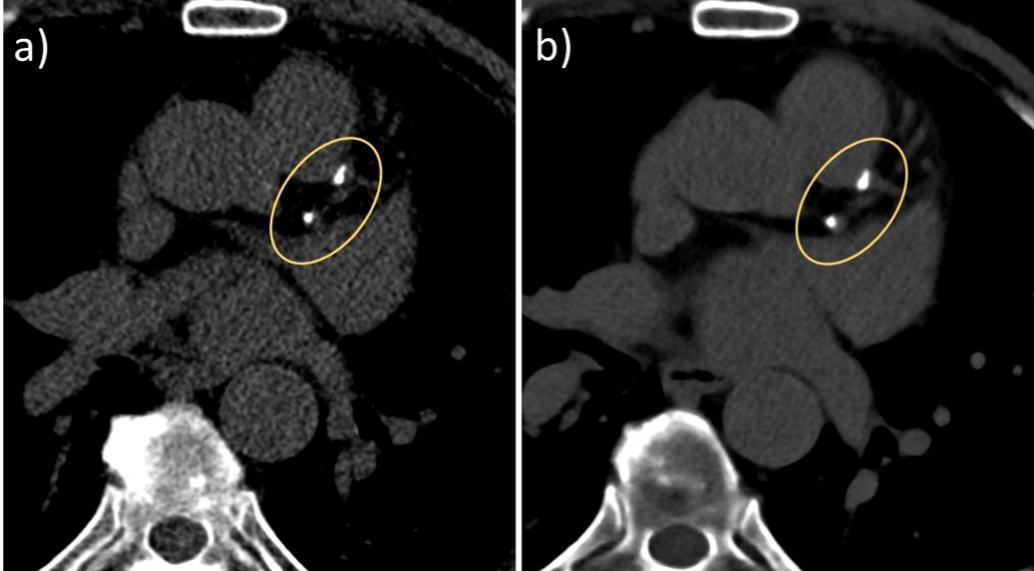
Clinical example of CAC scoring with PCD-CT. (**a**) Non-contrast CAC scoring scan of a 73-year-old male patient (120 kVp, 70 keV, 3 mm slice, Qr36, 61 bpm). Agatston score 322. (**b**) VNI image from a contrast-enhanced scan (120 kVp, 70 keV, 3 mm slice, Qr36, 57 bpm). Agatston score 330. Note the reduced image noise in the VNI image because of the higher radiation dose of the contrast-enhanced scan. CAC, coronary artery calcium; PCD-CT, photon-counting detector CT; VNI, virtual non-iodine.

Attempts have been made to determine CAC scores from spectral cCTA scans to avoid the non-contrast CAC scoring scan. CAC scores were measured on virtual non-contrast (VNC) images generated from two-material decomposition into iodine and soft tissue.^35–37^ This approach resulted in a good correlation, but systematic underestimation of the CAC scores—calcium is attributed to both base material images and appears in the VNC image only partly. Recently, a new technique based on two-material decomposition into iodine and calcium has been proposed for PCD-CT. It aims to remove iodine from contrast-enhanced cardiac CT images and leave only calcium in virtual non-iodine (VNI) images. These can be used for CAC scoring because calcium density should be preserved (Figure 2). With a moving coronary artery phantom, the accuracy of VNI-based CAC quantification using the Agatston method was demonstrated even at high heart rates up to 80 bpm.^38^ However, VNI-based scores decreased with increasing in-vessel attenuations. A study with 67 patients scheduled for cCTA on a clinical PCD-CT demonstrated high correlation, but still underestimation of the CAC scores for VNI images at 70 keV, albeit much lower than for VNC reconstructions.^39^ In a study with 90 patients undergoing late enhancement cardiac CT scans for transcatheter aortic valve replacement (TAVR) planning,^40^ CAC scores on VNI images at 70 keV with highest level iterative reconstruction (Quantum Iterative Reconstruction QIR4, Siemens Healthcare, Germany) showed excellent agreement to CAC scores on true non-contrast images. Aortic valve calcium (AVC) and mitral annular calcium (MAC) were best quantified at 80 keV regardless of QIR-strengths. Differences to the results of Emrich et al^39^ were attributed to a higher mean CAC score, a newer software version and lower in-vessel attenuation on late enhancement images compared with cCTA images. Subsequently, fine-tuning of keV and iterative reconstruction strength to improve scoring results with VNI images from cCTA was investigated.^41^

## Coronary CT angiography

Coronary CTA has emerged as a non-invasive alternative to cardiac catheterization and is now recommended in guidelines.^42^ Spatial resolution is still a challenge for cCTA in patients with severe coronary calcifications or with smaller coronary stents.^43,44^ Blooming and unclear visualization hamper the assessment of coronary plaque volume and plaque composition as well as the identification of adverse plaque characteristics.^45^

First benefits of increased spatial resolution with PCD-CT were seen in phantom experiments and small animal scans, demonstrating better imaging of coronary stents and superior in-stent lumen delineation,^46–52^ better visualization of coronary plaques^53,54^ and improved quantification accuracy of stenoses caused by calcified lesions.^55^ Although these studies were technically oriented, they did highlight opportunities as well as challenges (*e.g.* the increased image noise associated with high-spatial-resolution scans) for later patient scans.

An initial study of 14 human subjects undergoing cCTA on both a conventional CT and a PCD-CT prototype showed better image quality at comparable radiation dose for PCD-CT, with a significant reduction of blooming artifacts on coronary calcified plaques and higher diagnostic confidence scores, attributed to higher spatial resolution.^56^ In a study of eight patients with coronary stents, measured external stent diameters were smaller and internal stent diameters were larger with PCD-CT than with conventional CT, with reduced blooming artifacts and higher subjective image quality scores.^57^

The feasibility of cCTA with clinical dual source PCD-CT was evaluated in 92 patients.^58^ High image quality and CNR with 95% accessibility of coronary segments was achieved at a low median DLP of 90.9 mGy cm, corresponding to an effective dose of 1.4 mSv. The accessibility of coronary segments was limited by high amounts of CAC (Agatston score >600) or high heart rates in high-pitch mode.

**Figure 3.**
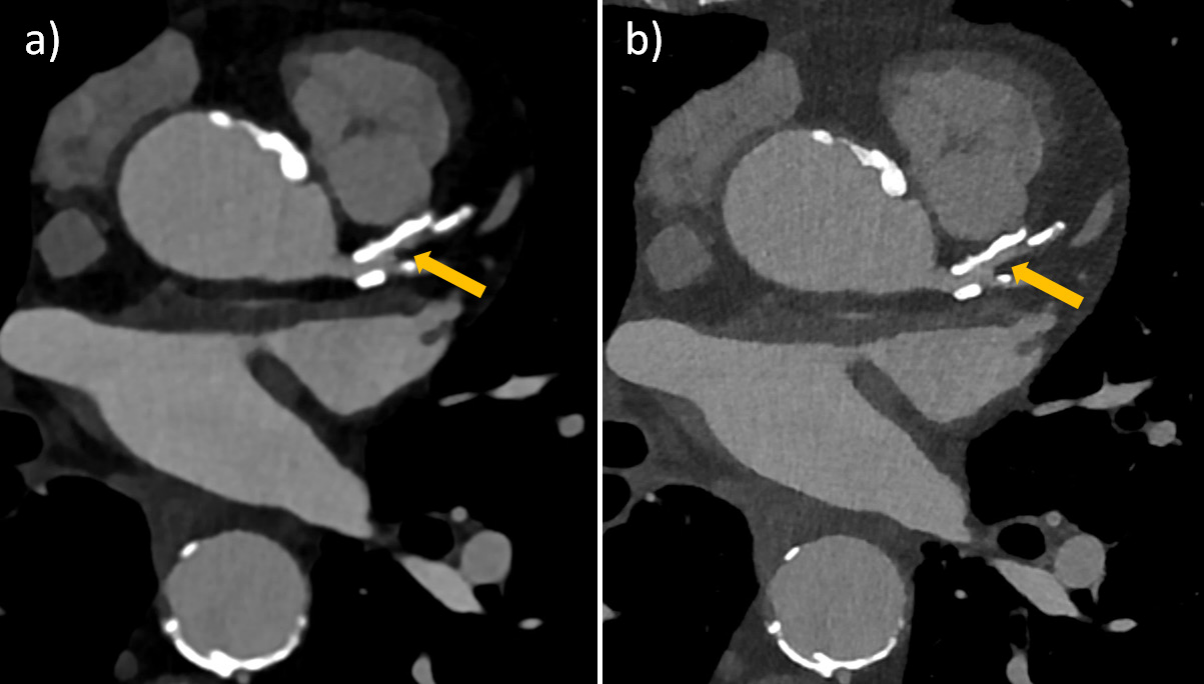
Ultra-high-resolution cCTA of an 82-year-old male patient on a clinical PCD-CT. Heart rate during data acquisition: 66 bpm. (**a**) Standard reference reconstruction (0.6 mm slice, Bv40, QIR4). (**b**) UHR reconstruction (0.2 mm slice, Bv56, QIR4). Note the improved visualization and larger perfused diameter (arrow) of the proximal LAD in the UHR image, which appears almost occluded in the standard reconstruction. cCTA, coronary CT angiography; PCD-CT, photon-counting detector CT.

**Figure 4.**
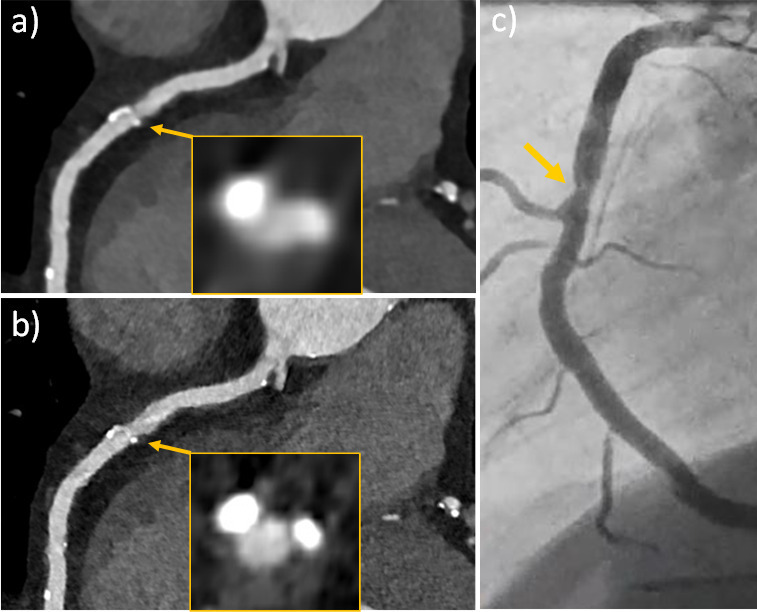
Ultra-high-resolution cCTA of a 62-year-old male patient on a clinical PCD-CT, prior to transcatheter aortic valve replacement. Heart rate during data acquisition: 74 bpm. (**a**) Standard reference reconstruction (0.6 mm slice, Bv40, QIR4). A moderate stenosis (60% in diameter) is seen in the proximal RCA (arrow). (**b**) UHR reconstruction (0.2 mm slice, Bv60, QIR4). Note the reduced calcium blooming, leading to a re-classification to a mild stenosis (38% in diameter). (**c**) Invasive catheter coronary angiography confirmed a mild stenosis in the proximal RCA (arrow). cCTA, coronary CT angiography; PCD-CT, photon-counting detector CT.

**Figure 5.**
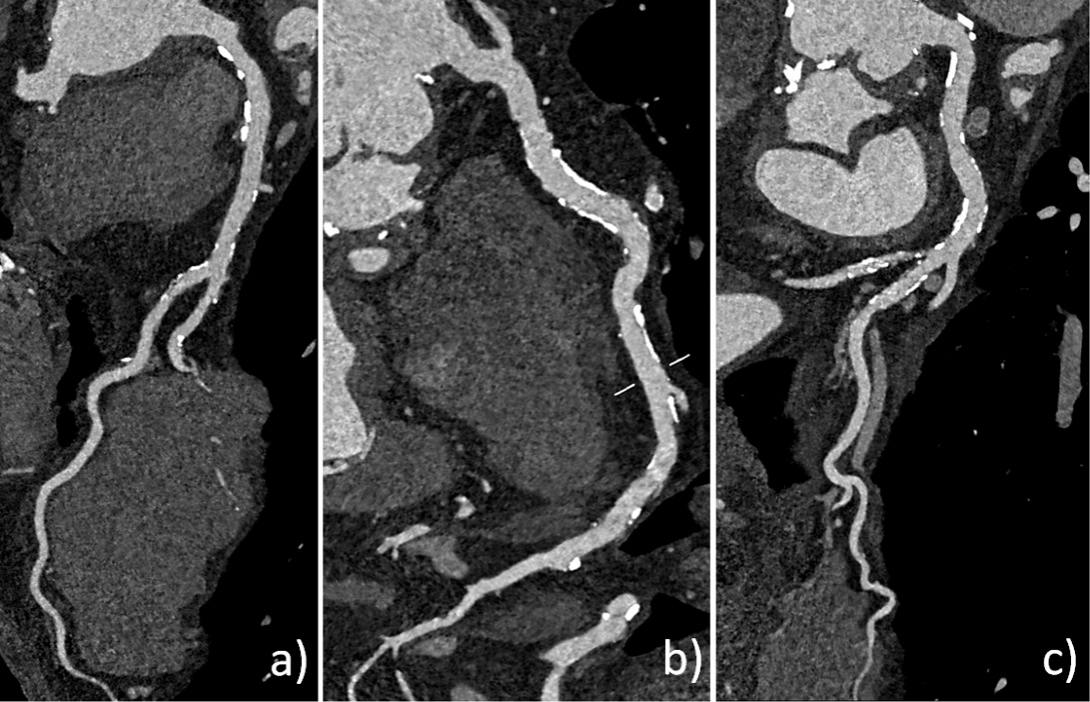
Ultra-high-resolution cCTA of a 68-year-old male patient on a clinical PCD-CT (retrospective ECG-gating). Heart rate during data acquisition: 72 bpm. UHR reconstruction (0.2 mm slice, Bv60, QIR4). (**a**, **b** and c) curved multiplanar reformations of the LAD, RCA, and CX allow reliable exclusion of significant coronary stenosis despite a very high CAC score of 5220. CAC, coronary artery calcium; cCTA, coronary CT angiography; PCD-CT, photon-counting detector CT.

In a study of 20 patients undergoing UHR-cCTA on a clinical PCD-CT, the optimal image reconstruction parameters were systematically evaluated.^59^ Excellent coronary plaque characterization and delineation of the adjacent vessel lumen with no perceived blooming was achieved with 0.2 mm slice width and image reconstruction with a sharp kernel (Bv64 or Bv72). Follow-up studies investigated the applicability of these results to coronary stent imaging^60^ and quantitative plaque analysis^61^ with UHR-cCTA. In 22 patients with 36 coronary stents,^60^ qualitative scores for stent evaluation increased from suboptimal/good for the reference standard (0.6 mm/Bv40) to very good/excellent for UHR reconstruction as described above. Absolute differences of measured *in-vitro* stent diameters decreased significantly from 1.2 ± 0.4 mm for the reference standard to 0.5 ± 0.3 mm for UHR reconstruction. In 22 coronary plaques of 20 patients,^61^ significantly lower total plaque volumes (reduction by 23%) and significantly lower calcified plaque components (reduction by 32%) were found on UHR reconstructions as compared with reference standard reconstructions. The volume of fibrous components remained similar, while that of low-attenuating components increased significantly. In a case study of a 68-year-old male patient, UHR-cCTA with clinical PCD-CT could visualize microcalcifications and fibrous caps confirmed by optical coherence tomography (OCT).^62^ Low attenuation and microcalcifications are indicative of high-risk plaques.^63^ Thus, UHR-cCTA can potentially improve patient risk stratification.^61^ Figures 3–5 show clinical examples of UHR-cCTA.

The importance of high temporal resolution for UHR-cCTA was demonstrated in 30 patients.^64^ Subjective image quality of the coronaries was significantly superior, stent blooming artifacts were significantly lower and image sharpness was higher in reconstructions with a temporal resolution of 66 ms as compared to 125 ms. In a study with 68 patients with clinically indicated cCTA prior to TAVR, UHR-cCTA on a clinical PCD-CT provided high diagnostic accuracy in the detection of CAD compared with invasive catheter angiography (ICA) as the gold-standard.^65^ Accuracy was 83% for patients with severe coronary calcifications (Agatston score >1000) and 93% for patients with prior stent implantation. According to the authors, ICA could have been avoided in 54% of the participants, signifying a potential benefit of UHR-cCTA.

**Figure 6.**
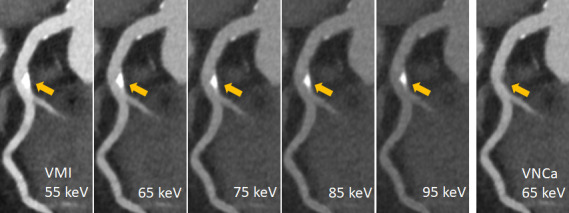
Spectral cCTA of a 73-year-old patient on a clinical PCD-CT (0.6 mm slice, Qr40, QIR3, 55bpm). Calcified plaque in the LAD (arrow). VMIs at 55, 65, 75, 85 and 95 keV: note the visually perceived reduction of Ca-blooming with higher keV at the expense of reduced iodine contrast. VNCa at 65 keV: the calcified plaque is removed from the contrast filled vessel without reducing the iodine contrast. cCTA, coronary CT angiography; PCD-CT, photon-counting detector CT.

With clinical PCD-CT, spectral information can so far only be exploited in standard scan mode at reduced spatial resolution compared to UHR-cCTA. In standard scan mode, accurate spectral material decomposition at high temporal resolution and in high-pitch spiral scans was confirmed in phantom scans,^66^ as well as stability of spectral results among different acquisition modes and heart rates.^67^ With VMI reconstructions imaging results can be fine-tuned to clinical requirements—low keV VMIs increase image contrasts in cCTA, while high keV reconstructions reduce blooming of calcified plaques. This has already been observed with conventional spectral CT scanners^68,69^ but has not been routinely exploited for cCTA. In a study of eight subjects undergoing cCTA on a prototype PCD-CT and on conventional spectral CT,^70^ better detectability of the coronary lumen, higher vessel sharpness and overall quality were observed for 40–90 keV VMIs of the PCD-CT. The difference was most pronounced at low keV. In a combined phantom and patient study on a clinical PCD-CT,^71^ improved CNR, vessel sharpness, and vessel attenuation were obtained with VMI reconstructions at low keV with high levels of iterative reconstruction, with best results at 40 keV and QIR4. The increased iodine CNR in VMIs at low keV may allow a reduction in the amount of contrast agent administered. In a phantom study, diagnostic quality for cCTA was still obtained at 50% contrast media concentration in the same 50 ml bolus when VMIs at 40 keV were used for evaluation.^72^ A recent study in 100 patients showed that contrast media can be reduced by up to 40% while maintaining a diagnostic image quality of cCTA because of the high iodine CNR of PCD-CT in VMIs at 45 keV.^73^ In a group of 53 patients undergoing cCTA in the high-pitch spiral scan mode of a clinical dual source PCD-CT with either standard contrast dose or reduced contrast dose, half the standard contrast dose (30 ml of a 350 mg I/mL contrast agent instead of 60 ml) was sufficient to achieve diagnostic image quality in VMIs at 50 keV.^74^ Additional evaluation of 100 keV VMIs improved the reader confidence in the assessment of luminal stenosis both in the presence of calcified plaque and within a stent. The combined evaluation of 50 keV and 100 keV VMIs had a diagnostic impact, downgrading the CAD-RADS classification in 6 patients. The lower CAD-RADS classification was attributed to decreased calcium blooming in 100 keV images (Figure 6).

A new application based on spectral two-material decomposition into calcium and iodine aims to remove calcified plaques from the contrast filled vessels without reducing the iodine contrast in a virtual non-calcium (VNCa) image. This application has so far been validated in a phantom study^75^ demonstrating reduced blooming artifacts from heavily calcified plaques and improved image interpretability at simulated heart rates up to 80 beats per minute. However, a possible better assessment of the coronary artery lumen must be proven in clinical studies. Figure 6 shows a clinical example.

## Evaluation of the myocardium and other applications

Assessing the myocardium is left to cardiac MR or nuclear imaging in clinical routine. Attempts have been made to use CT to determine ischemia or permanent scar, *e.g*. by static or dynamic cardiac CT perfusion,^76,77^ or by late enhancement imaging.^78^ The acquisition of spectral data can bring incremental improvements through better quantification.

**Figure 7.**
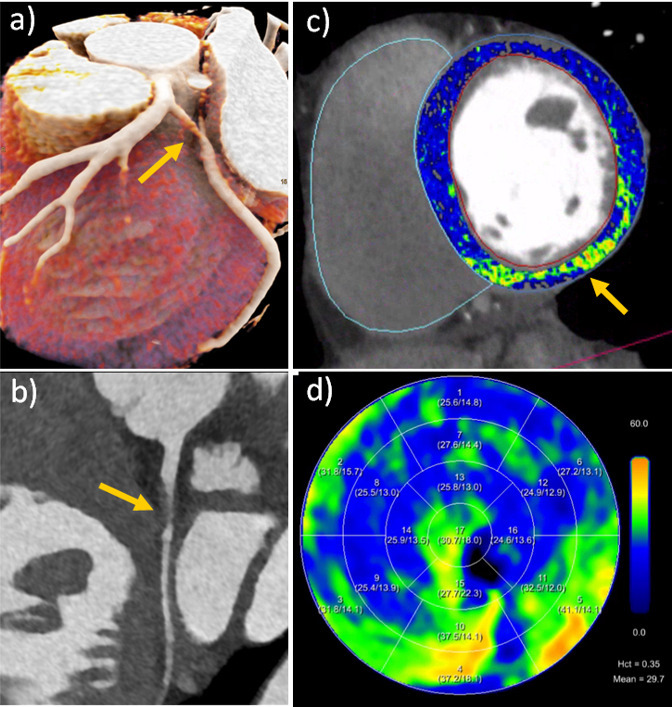
Spectral cCTA and ECV-evaluation of a 46-year-old female patient on a clinical PCD-CT (144 × 0.4 mm, 0.6 mm slice thickness, Bv40, QIR 3, 57 bpm). (**a, **and b) Cinematic rendering and curved planar reformation from cCTA show a spontaneous dissection of the proximal CX artery (arrow). (**c**) Overlayed color map from late enhancement shows a focal area of increased ECV-fraction (arrow) in the inferior wall, corresponding to the CX dissection in this left dominant coronary system. (**d**) Polarmap of the ECV-distribution. cCTA, coronary CT angiography; ECV, extracellular volume; PCD-CT, photon-counting detector CT.

CT late enhanced images are acquired 5–10 min after the administration of iodinated contrast agent for a cCTA.^78^ Myocardial fibrosis, mainly due to myocardial infarction, but also due to dilated and hypertrophic cardiomyopathies, amyloidosis, myocarditis, and sarcoidosis, causes hyper-enhancement in late CT scans. Instead of pure visual assessment, the extracellular volume (ECV) fraction can be calculated^79^ which indicates the percentage of extracellular matrix in the whole myocardium. To determine ECV with CT, the change in iodine concentration in the myocardium is normalized by the respective change in the blood pool, either by subtracting the images of a pre-contrast scan from a late enhanced scan,^80,81^ or by calculating quantitative iodine maps from a late-enhanced spectral CT scan.^82^ The feasibility of ECV-determination with PCD-CT was demonstrated in a study of 30 patients with severe aortic stenosis scheduled for TAVR. All patients underwent a non-enhanced CAC scoring scan, a cCTA, and a late scan 5 min after administration of 100 ml of a 370 mg I/mL contrast agent.^83^ ECV fractions were calculated with both the subtraction method and the spectral method and showed high correlation between each other. The medium ECV fraction was 30.5%, in good agreement with literature values for patients with severe aortic stenosis.^84,85^ Two focal ECV elevations correlated with known previous myocardial infarctions. In another study with 29 participants who underwent same-day cardiac PCD-CT and MR, ECV-values were compared with MR representing the standard of reference.^86^ PCD-CT showed strong correlation with MR for midventricular and global ECV quantification, but a slight overestimation of ECV by approximately 2% for the spectral method and a slight underestimation by 3% for the subtraction method. The detection and exact delineation of myocardial scars is important in patients with arrhythmia to plan ablation procedures. Such patients often have contraindications for MR imaging, and PCD-CT with acquisition of a late enhancement phase can serve as an alternative in these patients.^87^ An image example of late enhancement and ECV quantification is shown in Figure 7.

The amount of epicardial adipose tissue (EAT) is directly related to the development and severity of a variety of cardiovascular and metabolic diseases.^88^ Determination of EAT volume from cardiac CT scans without and with contrast agent is possible based on CT-number thresholds, but these depend on the X-ray tube voltage and the presence of contrast agent,^89^ and a standardized approach is still lacking. PCD-CT could provide standardization in this regard. Reconstruction of VMIs at a fixed keV level can provide fat attenuation values independent of X-ray tube voltage. It was found that VMIs at 70 keV gave the best approximation of fat attenuation measured by conventional CT at 120 kVp.^90^ For contrast-enhanced scans with PCD-CT, both VNC images and calcium-preserving VNI images were used to quantify the amount of EAT,^91^ with VNI images resulting in a negligibly small difference to TNC (−3%).

The performance of PCD-CT in examining patients prior to and post-TAVR was evaluated in a case series, showing advantages in assessing the aortic valve, aortic root, coronary arteries and possible vascular access routes through ultra-high spatial resolution and spectral capabilities.^92^

In children undergoing high-pitch contrast-enhanced CT because of suspected congenital heart defects, PCD-CT offered higher cardiovascular imaging quality than conventional dual source CT at similar radiation dose (0.50 *vs* 0.52 mSv).^93^

## Discussion

We investigated the potential and current uses of PCD-CT in cardiology based on the literature published to date, which includes applications such as CAC scoring, cCTA, myocardial assessment, and others.

In CaC scoring, spectral data acquisition with reconstruction of VMIs reproduces well the established results of conventional CT while reducing radiation dose. A reduction in mean DLP by 26%, from 45 mGy cm for conventional CT to 33.4 mGy cm for clinical PCD-CT, was reported.^30^ Depending on the protocol used for conventional CT, dose savings can be as high as 50%.^27^ Calculation of CAC scores in VNI images from contrast-enhanced scans may lead to radiation dose reduction by eliminating the non-contrast CAC scoring scan.

For cCTA, the greatest advantage of PCD-CT over conventional CT appears to be the acquisition of ultra high-resolution data, with the potential to expand the scope of cCTA. Initial evidence suggests UHR-cCTA may replace diagnostic ICA even in patients with severe calcifications and with prior stent implantation, thereby changing clinical management.^65^ UHR-cCTA supports current research topics such as coronary plaque analysis by providing more refined and potentially more accurate information, which may lead to improved patient risk stratification.^61,62^

When acquiring spectral data, cCTA image results can be flexibly adapted to clinical requirements through the reconstruction of VMIs—be it to reduce Ca blooming or metal artifacts with high keV or to increase suboptimal vessel contrasts or possibly even use less contrast agent with low keV.^71–74^ These practical advantages are helpful in routine diagnosis, and they may reduce the number of scans with limited diagnostic value. Refined spectral image analysis techniques, *e.g.* to remove calcified plaques from the contrast-enhanced coronaries, cannot yet be meaningfully assessed due to the lack of studies to date.

While the radiation dose of cCTA with PCD-CT at standard resolution is comparable to dose-optimized examinations with conventional CT (*e.g.* DLP = 90.9 mGy cm, corresponding to 1.4 mSv in an initial multicenter study),^58^ higher radiation dose has been reported for ultra high-resolution examinations (*e.g.* DLP = 936 mGy cm, corresponding to 13.3 mS).^65^ In CT, higher spatial resolution is generally associated with higher radiation dose. However, the diagnostic gain from the UHR examination should be considered as well, and the radiation dose can be further optimized: Hagar et al^65^ used dose modulation with ECG pulsing set at 20–80% of the R–R interval. An adjustment of this relatively wide window may lead to lower radiation dose in future studies. Lower radiation dose values were already reported in Mergen et al.^59^

Evaluation of the myocardium benefits most from spectral imaging with PCD-CT which allows for iodine quantification to characterize myocardial perfusion. The applications are diverse, be it the determination of perfusion defects in first-pass enhancement scans as additional information to cCTA, or the visualization of defects in late-enhancement scans including ECV quantification.^83,86^ PCD-CT is not yet widely available—there is one commercially available system from one manufacturer, other manufacturers have so far only installed preclinical prototypes. They all share, however, a focus on high spatial resolution and spectral capabilities as key requirements for cardiac CT, although they are realized with different approaches. The commercial system also offers very good temporal resolution through the dual source principle.^64^

PCD-CT is not a new modality but rather an evolution and refinement of conventional CT. It will not take over the specific applications of other imaging modalities, such as MR imaging, and make them obsolete. In particular, the lack of radiation dose, the high tissue contrast and the multiparametric capabilities of MR imaging will remain outstanding. However, initial findings suggest that the PCD technology may still offer significant improvements in cardiac CT. Superior diagnostic capabilities, reduced need for re-imaging, and potential prevention of unnecessary procedures, *e. g*. diagnostic ICA even in high risk patients,^65^ may outweigh the current high acquisition costs and contribute to the widespread use of PCD-CT in a cost-oriented healthcare environment.

## Outlook

Refined image analysis based on radiomics, and deep learning are increasingly used in cardiac CT,^94^ *e. g*. for assessment of EAT^95^ and for characterization of coronary artery plaques.^96^ The goal is to predict the risk of adverse events. The higher spatial resolution of PCD-CT, combined with additional spectral information, may lead to significant advances in this area. Organic samples could be better differentiated by their radiomics features in high-resolution PCD scans,^97^ and PCD-CT provided high test–retest stability of features which may facilitate the implementation of radiomics analysis in clinical routine.^98^ In first clinical studies, radiomics features of the myocardium were compared between PCD-CT and conventional CT.^99^ Texture changes of both periaortic adipose tissue^100^ and left ventricular myocardium^101^ were found to be associated with the severity of CAC. Using 100 keV VMIs and VNC images derived from coronary PCD-CTA, low- and high-risk coronary plaques could be automatically differentiated by a machine-learning approach based on their radiomics features.^102^ We are only at the beginning of a development here the full scope of which remains to be seen.
